# A specific, promoter-independent activity of T7 RNA polymerase suggests a general model for DNA/RNA editing in single subunit RNA Polymerases

**DOI:** 10.1038/s41598-018-32231-6

**Published:** 2018-09-17

**Authors:** Subha Narayan Sarcar, Dennis L. Miller

**Affiliations:** Department of Biological Sciences, The University of Texas at Dallas, Richardson, Texas 75083-0688 USA

## Abstract

Insertional RNA editing has been observed and characterized in mitochondria of myxomycetes. The single subunit mitochondrial RNA polymerase adds nontemplated nucleotides co-transcriptionally to produce functional tRNA, rRNA and mRNAs with full genetic information. Addition of nontemplated nucleotides to the 3′ ends of RNAs have been observed in polymerases related to the mitochondrial RNA polymerase. This activity has been observed with T7 RNA polymerase (T7 RNAP), the well characterized prototype of the single subunit polymerases, as a nonspecific addition of nucleotides to the 3′ end of T7 RNAP transcripts *in vitro*. Here we show that this novel activity is an editing activity that can add specific ribonucleotides to 3′ ends of RNA or DNA when oligonucleotides, able to form intramolecular or intermolecular hairpin loops with recessed 3′ ends, are added to T7 RNA polymerase in the presence of at least one ribonucleotide triphosphate. Specific ribonucleotides are added to the recessed 3′ ends through Watson-Crick base pairing with the non-base paired nucleotide adjacent to the 3′ end. Optimization of this activity is obtained through alteration of the lengths of the 5′-extension, hairpin loop, and hairpin duplex. These properties define a T7 RNAP activity different from either transcriptional elongation or initiation.

## Introduction

RNA editing is an alteration of the genetic information in RNA relative to its DNA template. It is an additional step in gene expression, where specific nucleotides at specific positions are substituted for, added to, or deleted from the RNA in order to produce functional RNAs, especially mRNAs with a functional open reading frame. The term “RNA editing” was introduced in 1986 to describe the insertion of four uridine nucleotides into specific sites of mitochondrial cox2 mRNA in Trypanosome mitochondria^1^. There are two major forms of RNA editing, [A] substitutional, involving nucleotide modification, typically a transamination or deamination, which converts one nucleotide to another, for example, editing of tRNAs in *Acanthamoeba castellanni*^2^ or single nucleotide conversions such as A to I^3^), C to U or U to C^4^ and [B] insertional in which a nucleotide (or nucleotides) are inserted or deleted to produce a functional RNA^5–7^.

Two different types of insertional editing have been observed, post-transcriptional and co-transcriptional, each exemplified by a mitochondrial DNA system in which that type of insertional RNA editing is used extensively to produce functional mitochondrial RNAs. Post-transcriptional RNA editing, as exemplified by RNA editing in the mitochondria of trypanosomes, requires an endonuclease to break the RNA backbone at a specific site, a terminal transferase to add a nucleotide or nucleotides to the 3′ end of the RNA at the break site, and an RNA ligase to restore the RNA backbone at the site of the insertion or deletion^8–12^. Specificity of the nucleotide insertion or deletion site and the number of nucleotides inserted or deleted at a site is determined by an antisense RNA, termed a guide RNA [gRNA], which has the genetic information to produce functional mRNAs significantly different from the template DNA^13^.

Co-transcriptional insertional RNA editing as exemplified by RNA editing in the mitochondria of myxomycetes such as *Physarum polycephalum* produce functional mRNAs, rRNAs, and tRNAs^7,14–17^ different from their mtDNA template through non-templated addition of specific nucleotides at specific sites in nascent RNAs by the mitochondrial RNA polymerase (mtRNAP)^15,18–20^. Two major questions in the mechanism of this type of editing are: [A] How is the correct nucleotide inserted without a DNA template? [B] How is the correct site of insertion determined? Miller & Miller^20^ and Visomirski-Robic & Gott^19^ have each proposed that an unknown mechanism causes the mitochondrial RNA polymerase to pause during elongation of RNA on the DNA template, and insert a specific, non-templated nucleotide (or occasionally nucleotides), before continuing DNA-templated elongation. Miller & Miller^20^ have shown that the Physarum mtRNAP is able to add nucleotides to the 3′ end of RNAs in the absence of DNA *in vitro*. However, with complex RNA populations without DNA templates *in vitro* any of the four ribonucleotides can be added.

Both aspects of the specificity of this co-transcriptional RNA editing involve the mtRNAP. Mitochondrial RNAPs are single subunit polymerases^21^ found in essentially all eukaryotic organisms and have conserved structural and functional similarities that indicate that they evolved from a common ancestor present at or near the advent of the eukaryotic line^18^. Interestingly, the conservation of these structural and functional similarities extend to other single subunit polymerases, such as the bacteriophage RNAPs^22–24^, DNA polymerases (DNAPs) such as the Klenow fragment of DNAP I in *E. coli*^25–27^, and reverse transcriptases (RNA-directed DNA polymerases) such as the HIV-1 reverse transcriptase^28–30^, constituting a super-family of single subunit polymerases with structural and functional homology.

The prototype RNA polymerase for this group of polymerases is the T7 RNAP encoded by the T7 *E. coli* bacteriophage DNA and it has been extensively studied^23,24,31,32^. The crystal structure of T7 RNAP has been solved along with the different domains and their functions corresponding to both initiation and elongation processes^33–35^. It is a single subunit ~99 kDa protein (883 amino acid)^36^, found in Bacteriophage T7 and is primarily involved in transcription activity without the need of any other accessory transcription factors. The specificity and robustness of T7 RNA polymerase’s transcription activity comes from its specificity towards its promoter, a 23 nucleotide dsDNA sequence^37–39^. This specificity towards the promoter, involves the promoter recognition loop^40^ as well as the N terminus domain in T7 RNA polymerase^41,42^. T7 RNA polymerase can successfully carry out transcription on a synthetic ssDNA oligonucleotide template containing double stranded T7 promoter sequence^43,44^.

In addition to *de novo* initiation which requires a promoter, single stranded DNA template, and the four ribonucleotide triphosphates; and elongation which requires a duplex with one strand having a recessed 3′ end (primer), the other strand 5′ extended (template), and four ribonucleotide triphosphates; there have been reports of a third activity in which addition of ribonucleotides occurs at the 3′ end of RNAs in the absence of a promoter or DNA template^45–48^. This activity was incorrectly attributed to the primer extension activity of elongation. Until recently, this activity of T7 RNAP has not been well characterized and most of the research on it has been focused on ways to prevent the addition of non-templated nucleotides to the 3′ ends of run-off RNAs^46,49,50^. Here we report the characterization of this activity. We show [A] that the T7 RNAP can add nucleotides to the 3′ end of RNAs and DNAs in a process that is neither elongation nor initiation, but is a type of RNA/DNA editing, [B] that the added nucleotide is added specifically as determined by base pairing of either an inter- or intramolecular template with an extended 5′ end relative to the recessed 3′ end, and [C] that the properties of the oligonucleotide that is provided determine the efficiency of nucleotide addition and distinguish this activity from either initiation or elongation activities. We will discuss this activity in terms of a general model of RNA editing by single subunit polymerases that may explain the specificity of nucleotide insertion during RNA editing by the *Physarum* mtRNAP.

## Material and Methods

### Oligonucleotides

Synthetic single stranded DNA oligonucleotides (10 nmol scale) were synthesized by Eurofin-Operon. Oligonucleotides were dissolved in water to 200 µM and stored at 4 °C.

### Oligonucleotide naming convention

Each oligonucleotide is named by a five number sequence separated by dashes where the first number is the oligonucleotide length, the second number is the length of the single stranded 5′ extension. The third and fifth number are the length of the double stranded region (stem of hairpin), and the fourth number is the length of the hairpin loop.

#### T7 RNAP, reaction buffer, ribonucleotide triphosphates

T7 RNA polymerase (New England Biolabs (NEB) catalog number M0251S) was supplied in: 100 mM NaCl, 50 mM Tris-HCl (pH 7.9), 1 mM EDTA, 20 mM 2-mercaptoethanol, 0.1% Triton X-100 and 50% glycerol and stored in a −20 °C freezer.

NEB 10 × RNA pol reaction buffer was supplied. 1 × RNA Pol Reaction Buffer contains 40 mM Tris-HCl, 6 mM MgCl_2,_ 2 mM spermidine, 10 mM dithiothreitol, pH 7.9 @ 25 °C, 1 mg/ml BSA (Bovine Serum Albumin).

#### Oligonucleotide radiolabeling

The *in vitro* oligonucleotide labeling protocol was derived from the *in vitro* transcription protocol recommended by New England Biolabs (NEB) for their T7 RNA polymerase^51^. For radiolabeling, (α-^32^P) rGTP, or (α-^32^P) rATP (3000 Ci/mmol 10 mCi/ml; Perkin Elmer, product numbers BLU006H and BLU003H, respectively) were used. *In vitro* oligonucleotide radiolabeling was assayed in reactions containing 6 µl of 200 µM ssDNA oligonucleotides, 2 µl 10 × RNA polymerase buffer, 2 µl of 5 mM ribonucleoside triphosphates, and 1 µl of α-^32^P radiolabeled rATP/rGTP to a final volume of 20 µl. The reactions were incubated at 37 °C water bath for 60 minutes. After incubation 2 µl of 6× loading dye (10 mM Tris-HCl (pH 7.6) 0.03% bromophenol blue, 60% glycerol, 60 mM EDTA) was added to individual reaction mixtures and samples were run on 15% TBE (Tris-Borate EDTA) polyacrylamide gels at a constant 200 V and 75 mA, until the bromophenol blue migrated three-quarters of the length of the gel. Experiments were routinely repeated on both denaturing and non-denaturing gels to check their reproducibility.

#### Detection and Quantification

Nucleic acids in the gels were stained with 1× TBE, Ethidium Bromide for 10 min and then visualized under Bio-Rad Gel Doc^TM^. Detection of incorporated radioactivity was achieved by exposure to a phosphor screen (Molecular Dynamics) and scanned on a Phosphor Imager (Storm 840, Amersham Biosciences). Quantification of the phosphor image was done using ImageQuaNT software version 5.2 (Molecular Dynamics). Only the most intense band in each lane was quantitated. Graphical representation of major band intensities below each gel are shown to allow easier interpretation.

## Results

**Figure 1:**
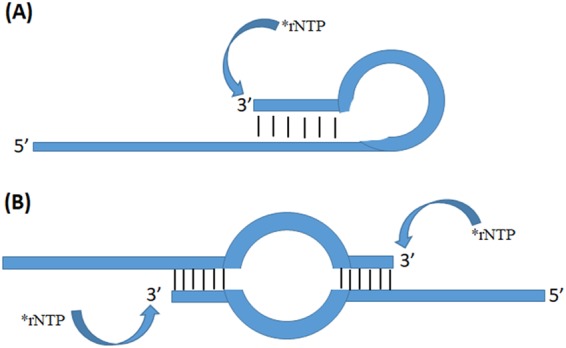
Diagram of the Intramolecular and Intermolecular base pairing forms in the oligonucleotides used in the study. (**A**) intramolecular base pairing form of the oligonucleotide showing the site of nucleotide addition. (**B**) intermolecular base pairing form of the oligonucleotide showing the sites of nucleotide addition.

Several laboratories have reported that single subunit RNAPs could add radiolabeled nucleotide triphosphates to the 3′ end of RNA in the absence of a DNA template^20,48,52^ and that T7 RNAP could add ribonucleotides to the 3′ end of DNA and RNA^44,46,47,53–55^. We hypothesized that these observations indicated that these RNAPs had a novel activity similar to the RNA editing observed in mitochondria of *Physarum polycephalum*^7^ and other myxomycetes^56^. To study this activity, we have developed an *in vitro* assay for labeling the 3′ ends of RNA and DNA oligonucleotides using T7 RNAP and a single radiolabeled ribonucleotide triphosphate. In contrast to using complex mixtures of RNA or DNA, with these simple oligonucleotide sequences, some oligonucleotides failed to label while others labeled efficiently. We have determined that this labeling specificity depends on the ability of the 3′ end of the oligonucleotide to anneal with an internal complementary sequence which positions the 3′ end next to a nucleotide that is not base paired in the 5′ extension of the oligonucleotide. This can take place within the same oligonucleotide (intramolecular base pairing) or between complementary sequences in two separate oligonucleotides (intermolecular base pairing) as shown in Fig. 1. Most of the oligonucleotides of this type exists in both forms in solution. Form B (Fig. 1B) can also be labeled, since it has two recessed 3′ ends.

**Figure 2:**
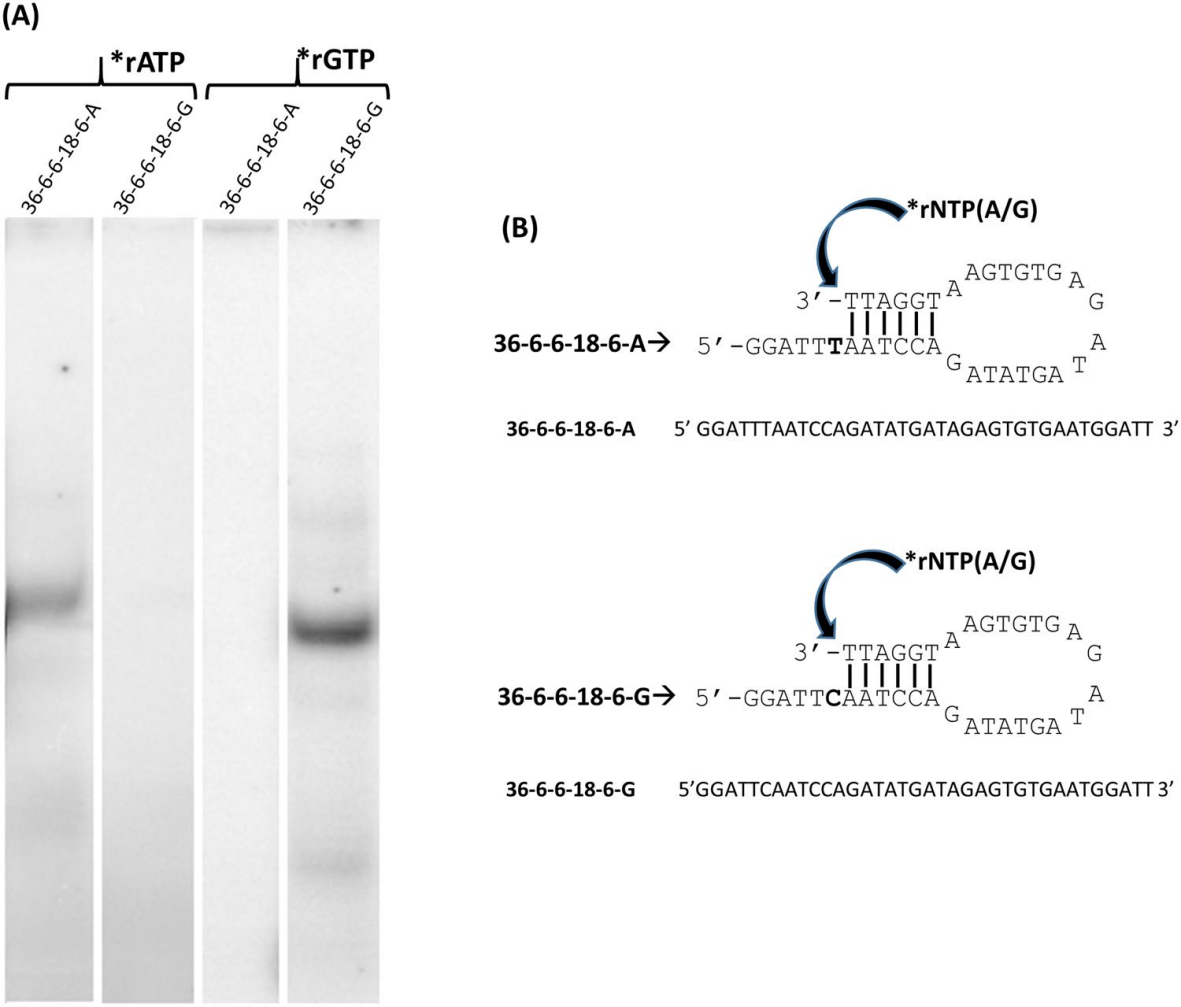
Specificity of oligonucleotide labeling by T7 RNA polymerase. (**A**) Autoradiograph of 15% polyacrylamide gel which shows 3′ end labeling by T7 RNAP on DNA oligonucleotides (36mer) in the presence of the α-^32^P GTP (right two lanes) or α-^32^P ATP (left two lanes). (**B**) The sequence and potential looped structure of the DNA oligonucleotides labeled in (**A**).

An example of these experiments is shown in Fig. 2. Two similar DNA oligonucleotides, 36-6-6-18-6-A and 36-6-6-18-6-G, each 36 nucleotides in length, and each with the potential to form intra or intermolecular duplexes of 6 base pairs with recessed 3′ ends (Fig. 2B), were incubated with T7 RNAP in the presence of either radiolabeled GTP or ATP. These oligonucleotides varied from one another by a single nucleotide in the first position of the 5′ extension template. Oligonucleotide 36-6-6-18-6-A has a T nucleotide in this position which will serve as a template for an A to be added to the 3′ double stranded end. Whereas oligonucleotide 36-6-6-18-6-G has a C nucleotide in the template position. After incubation the oligonucleotides were applied to a 15% polyacrylamide gel to separate the oligonucleotide from the unreacted radiolabeled nucleotides. Figure 2A shows an autoradiograph of the gel. Oligonucleotide 36-6-6-18-6-A was labeled with ATP, but not GTP, while oligonucleotide 36-6-6-18-6-G was labeled with GTP, but not ATP. Oligonucleotides similar to 36-6-6-18-6 but lacking the 6 base pair complementarity were not labeled (data not shown). Reactions with oligonucleotides and labeled nucleotides but in which the T7RNAP was omitted, were similarly negative (data not shown).

This experiment demonstrates that when presented with an oligonucleotide with a 3′ end with the potential to base pair as a recessed 3′ end, either inter- or intra- molecularly, the T7 RNAP is able to add the radiolabeled nucleotide to the 3′ end, but only if it can form a Watson-Crick base pair with the nucleotide in the 5′ extension (template) next to the nucleotide base paired with the 3′ end nucleotide (primer).

To better understand this self-templated labeling at the 3′ end and to optimize this reaction, the single stranded DNA oligonucleotides have been systematically altered to determine the importance and contribution of [1] the overall length, [2] the length and base composition of the potential duplex, [3] the length of the 5′ extension, and [4] loop length (number of nucleotides separating the complementary sequences) with the goal of optimizing the reaction.

### Contribution of 5′ extension length (V-V-6-8-6 series)

To determine the contribution of 5′ extension length, oligonucleotides were designed with increasing 5′ extension lengths (0–16 nucleotides), constant base pairing length (6 pairs), constant loop length (8 nucleotides), and increasing overall length (20–36 nucleotides).

**Figure 3:**
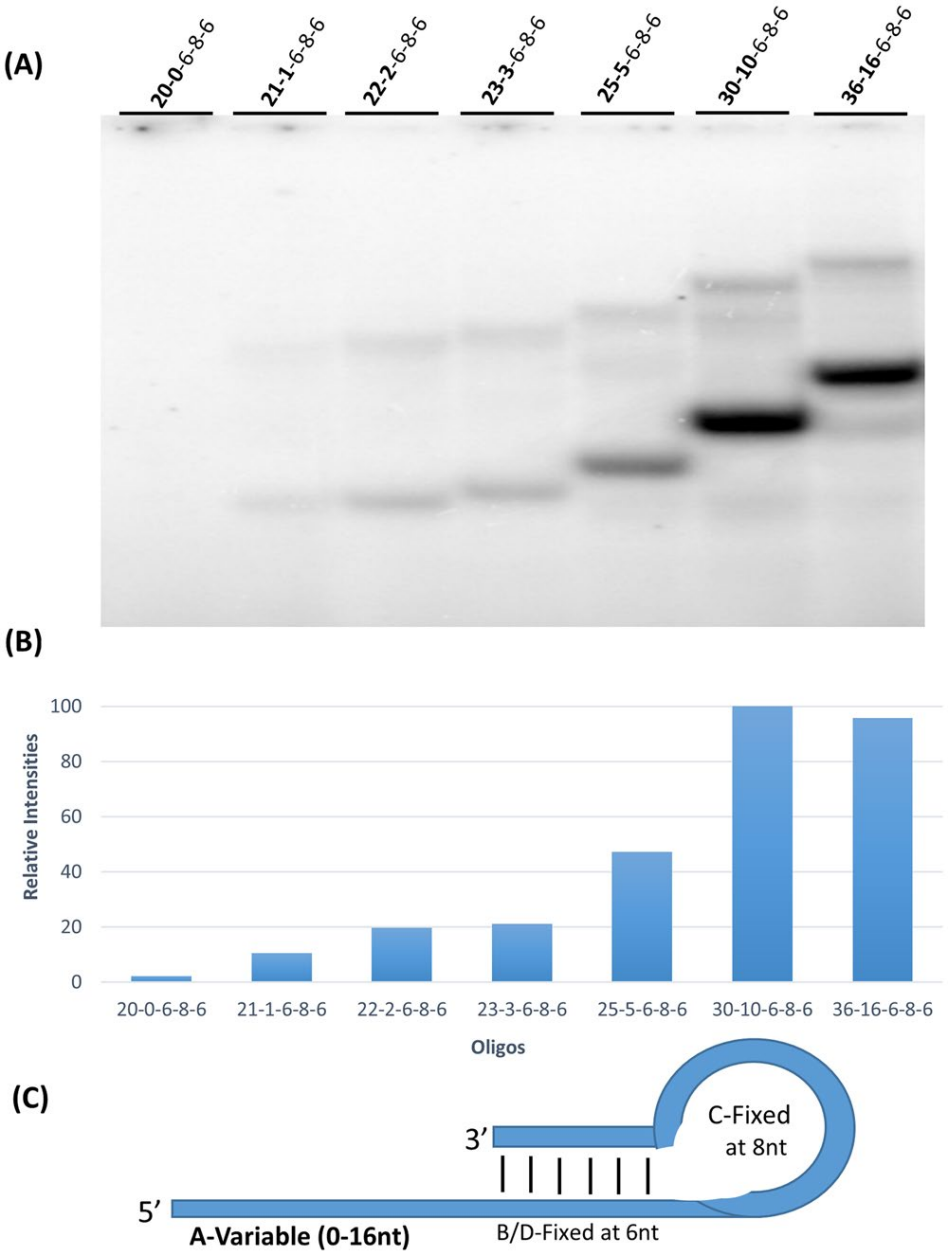
Effect of 5′-extension length variation on oligonucleotide labeling. (**A**) Autoradiograph of 15% polyacrylamide gel which shows 3′ end labeling by T7 RNAP on DNA oligonucleotides 36-0-6-8-6 to 20-16-6-8-6 in the presence of the α-^32^P ATP where there is a systematic increase of 5′ extension from 0 to 16 nucleotides. (**B**) Graphical representation showing the relative intensities of oligonucleotide labeling in the most intensely labeled (intramolecularly base paired) bands in each lane, normalized against the maximum intensity in the same gel. Minor low mobility (stable intermolecularly base paired) bands were not quantitated, but give consistent results with the intramolecularly base paired form. (**C**) The general intramolecular structure of the oligonucleotides used in (**A**) showing the number of nucleotides present in each feature of the hairpin loop and 5′ extension.

Figure 3 shows the results of these experiments. Oligonucleotide labeling was absent in the absence of a single stranded template (no 5′ extension, 20-0-6-8-6). Labeling increased with 1, 2, 3, and 5 nucleotide 5′ extensions. 5′ extensions of 10 and 16 nucleotides were most intensely labeled and were essentially equal in intensity. 5′ extensions from 1–10 nucleotides had essentially linearly increased labeling (a slope of 10 units per nucleotide extension length). Above a length of 10 nucleotides the labeling reached a plateau. Upper (lower mobility) bands in Fig. 3A show labeling of oligonucleotides with stable intermolecular base pairing to be proportional with lower (higher mobility) bands with intramolecular base pairing. In the 6-8-6 series oligonucleotide labeling increased with 5′ extension length up to about 10 nucleotides.

### Contribution of loop length (V-2-9-V-9 and V-2-6-V-6 series)

**Figure 4:**
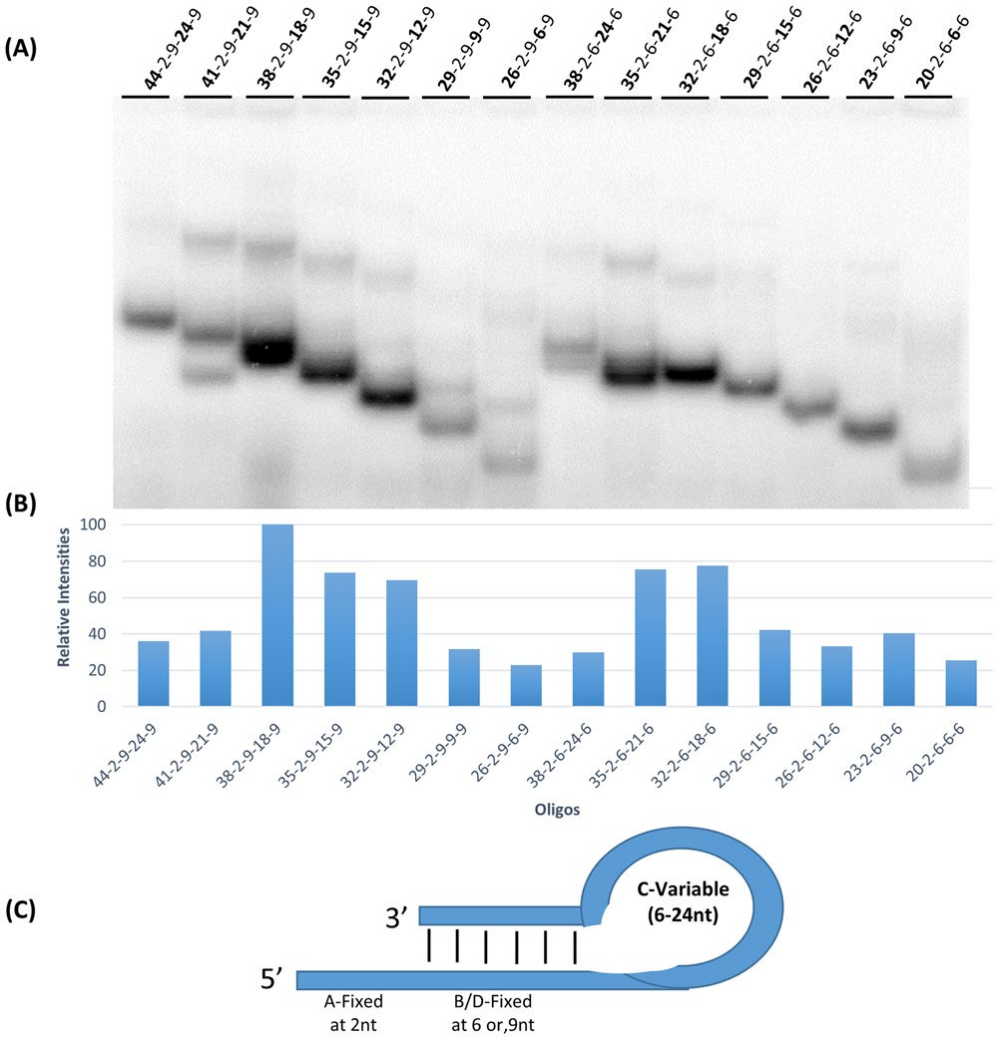
Effect of loop length variation on oligonucleotide labeling. (**A**) Autoradiograph of 15% polyacrylamide gel which shows 3′ end labeling by T7 RNAP on DNA oligonucleotides in the presence of the α-^32^P ATP where the loop length is systematically varied. (**B**) Graphical representation showing the relative intensities of the most intensely labeled bands in each lane (intramolecularly base paired). Minor bands corresponding to intermolecularly base paired oligonucleotides were not quantitated. (**C**) The general structure of the oligonucleotides used in (**A**) showing the number of nucleotides present in each feature of the hairpin loop and 5′ extension.

To determine the contribution of loop length to labeling efficiency, oligonucleotides were designed with constant 5′ extensions (2 nucleotides), constant double stranded regions (either 9 nucleotides or 6 nucleotides), and variable loop lengths (6–24 nucleotides) and variable overall length (20 nucleotides to 44 nucleotides). Nucleotide labeling efficiencies are shown in Fig. 4A.

Labeling is seen for oligonucleotides with both intramolecular and intermolecular base pairing. Figure 4B shows the relative intensities of the oligonucleotides with intramolecular base pairing. For oligonucleotides with a nine base pair double stranded duplex, the optimum labeling was seen in oligonucleotides with 12–18 nucleotide loops, loops longer or shorter than this were labeled less intensely. For oligonucleotides with a six base pair double stranded duplex, maximum labeling was seen for 18 to 21 nucleotides in the loop. Again, shorter or longer loop lengths labeled less well. Loop sizes for maximum labeling vary with the length of base pairing in the double stranded region. The smaller base pairing region (6 base pairs) gives optimum labeling with larger loop sizes (18–21 nucleotides) relative to the larger base pairing region (9 base pairs) which has optimum labeling with a smaller loop size (12–18 nucleotides). Overall length does not correlate with labeling intensity, for example, the two oligonucleotides that were 38 nucleotides in length gave dramatically different labeling intensities.

### Contribution of base paired region (32-2-V-V-V series, V-10-V-8-V series)

**Figure 5:**
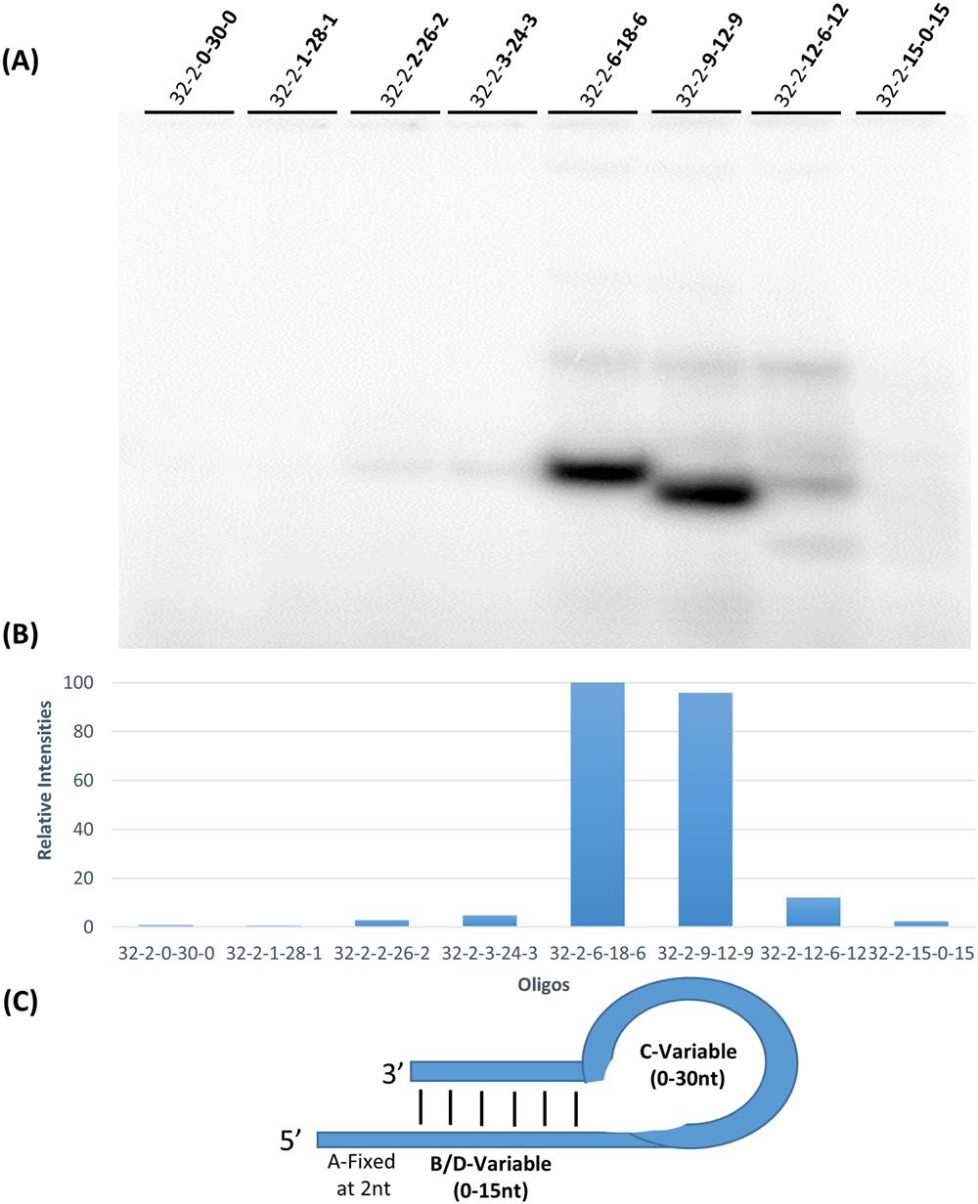
Effect of loop length variation and duplex length variation on oligonucleotide labeling. (**A**) Autoradiograph of 15% polyacrylamide gel which shows 3′ end labeling by T7 RNAP on DNA oligonucleotides in the presence of the α-^32^P ATP where the loop length and duplex length are systematically varied. (**B**) Graphical representation showing the relative intensities of oligonucleotide labeling with varying loop length after exposure, normalized against the maximum intensity in the same gel. Minor, intermolecularly base paired bands were not quantitated. (**C**) The general structure of the oligonucleotides used in (**A**) showing the number of nucleotides present in each feature of the hairpin loop and 5′ extension.

To determine the contribution of the length of the base pairing to 3′ end labeling efficiency, the base pairing length was systematically altered while keeping the overall length (32 nucleotides) and the 5′ extension length (2 nucleotides) constant (Fig. 5). To keep overall length constant, the loop length was decreased as the length of the base pairing region was increased. Oligonucleotides with 0, 1, 2, or 3 base pairing potential did not label efficiently, while base pairing regions of 6 or 9 base pairs (loop lengths of 18 nucleotides or 9 nucleotides respectively) did label efficiently. Surprisingly, oligonucleotides with the potential for even longer base pairing regions (12 and 15 base pairs) also did not label significantly. Again, overall length did not correlate with labeling efficiency.

**Figure 6:**
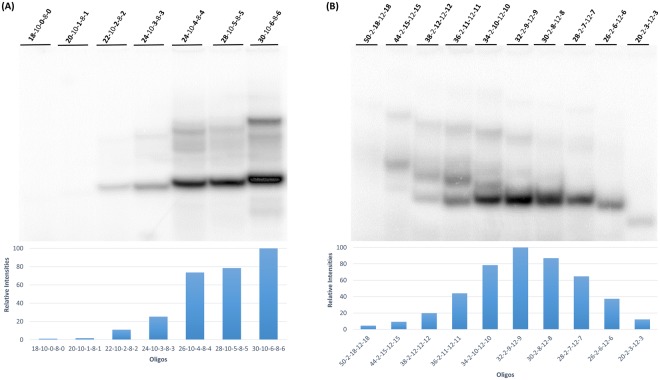
Effect of duplex length variation on oligonucleotide labeling. (**A**) Relative intensities of oligonucleotide labeling where duplex length is varied and where 5′-extension is fixed at 10 nucleotides and loop length is fixed at 8 nucleotides. (**B**) Relative intensities of oligonucleotide labeling where duplex length is varied and where 5′-extension is fixed at 2 nucleotides and loop length is fixed at 12 nucleotides. In both (**A**) and (**B**), the most intensely labeled (intra molecularly base paired) bands were normalized against the maximum intensity in the same gel. Minor low mobility (stable intermolecularly base paired) bands were not quantitated but appeared consistent with the intramolecular base paired form.

To further determine whether the contribution of the base pairing length to 3′ end labeling efficiency, we varied the length of the base pairing region while holding the length of the loop region (at either 8 nucleotides or 12 nucleotides) and the 5′ extension constant (at either 2 nucleotides or 10 nucleotides) constant. The V-10-V-8-V series is shown in Fig. 6A; the V-2-V-12-V series is shown in Fig. 6B. In Fig. 6A the duplex length is varied from 0 to 6 base pairs with constant 5′ extension (10 nucleotides) and constant loop length (8 nucleotides). Labeling efficiency increases with base pairing length. Maximum label efficiency is at 6 base pairs. In Fig. 6B the base paired region length is varied from 3 base pairs to 18 base pairs with constant 5′ extension (2 nucleotides) and loop length (12 nucleotides). Labeling efficiency increased with duplex length up to 9 base pairs but decreased with lengths greater than 9 base pairs to nearly zero at 18 base pairs. The 9 base pair sequence that gave the maximum labeling is 5′- TATAATATT-3′.

### Labeling efficiency of oligonucleotides with intermolecular base pairing

**Figure 7:**
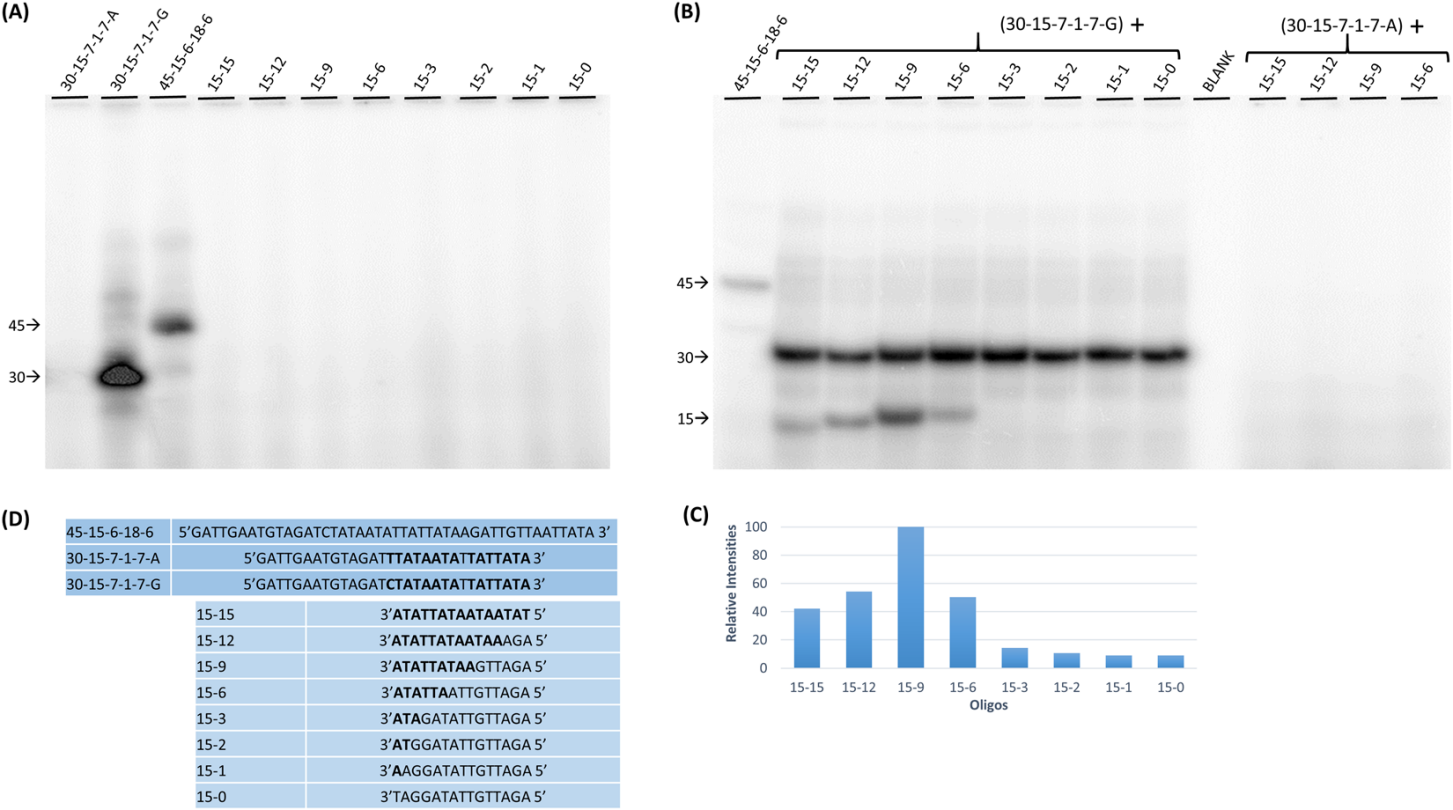
Oligonucleotide labeling with intermolecular duplex formation. (**A**) rGTP labeling of the individual oligonucleotides listed in (**D**). The numbers on the left are the size of oligonucleotides in nucleotides. (**B**) rGTP labeling of oligonucleotides in reactions involving a mixture of primer and template oligonucleotides. The left side of (**B**) shows the labeling of primers with various lengths of base pairing potential with template 30-15-7-1-7-G. The right side of (**B**) shows the labeling of primers with various lengths of base pairing potential with template 30-15-7-1-7-A. (**C**) Graphical representation showing the relative intensities of oligonucleotide primer labeling (with varying template base pairing potential) after exposure, normalized against the maximum intensity of the primer oligonucleotides in the same gel. (**D**) List of the sequences of the oligonucleotides used in this experiment. The upper three oligonucleotides are used as templates, while the lower eight oligonucleotides are used as primers.

To determine if this optimum base pairing length for labeling efficiency also pertained to intermolecular base pairing and to eliminate the contribution of loop length to labeling efficiency, a series of oligonucleotides with varying lengths of sequence complementary to a template oligonucleotide were designed. Figure 7D shows the sequences of these oligonucleotides. Oligonucleotide 30-15-7-1-7-G provides the 5′ extension nucleotide which serves as the template. It has the potential to form an intramolecular hairpin loop with a 7 base pair stem, 5′ extension, and recessed 3′ end with an adjacent non-base paired C nucleotide, or an intermolecular base pairing with itself with two 7 base pair regions each with a 5′ extension and a recessed 3′ end with an adjacent non-base paired C nucleotide. Oligonucleotide 30-15-7-1-7-A is identical to oligonucleotide 30-15-7-1-7-G except the non-base paired nucleotide next to the recessed 3′ end is a T instead of a C nucleotide. A third oligonucleotide 45-15-6-18-6 has the potential to form an intramolecular hairpin with a 6 base pair stem, 18 nucleotide loop, a 5′ extension, a recessed 3′ end with an adjacent non-base paired C nucleotide. This oligonucleotide serves as a positive labeling control when used with radiolabeled GTP.

Below the template strands in Fig. 7D are eight 15 nucleotide primer oligonucleotides. Oligonucleotide 15–15 is complementary to the 15 nucleotides on the 3′ end of both 30 nucleotide template oligonucleotides. The remaining 15 nucleotide primers retained 12 to 0 nucleotides of complementarity to the template at their 3′ end.

Figure 7A shows the labeling of each of the individual oligonucleotides by T7 RNAP in the presence of radiolabeled GTP. Both 45-15-6-18-6 and 30-15-7-1-7-G are labeled since they can form intramolecular or intermolecular base paired regions with a recessed 3′ end next to a C nucleotide that can base pair with GTP. 30-15-7-1-7-A is not labeled even though it can form the same base paired regions with a recessed 3′ end. However, in this case, the base adjacent to the 3′ end is an A nucleotide which cannot base pair with GTP. None of the 15 base pair primer oligonucleotides are labeled since they cannot form intermolecular or intramolecular base pairing regions with recessed 3′ ends.

Figure 7B shows the labeling of the oligonucleotides by T7 RNAP in the presence of radiolabeled GTP when the primer oligonucleotides are mixed with the template oligonucleotides in various combinations. The lanes of the left side of Fig. 7B show each of the eight 15 nucleotide primers mixed with 30-15-7-1-7-G. In each case 30-15-7-1-7-G is labeled indicating intramolecular or intermolecular base pairing with itself. In addition, primer oligonucleotides 15-15, 15-12, 15-9 and 15-6 are labeled indicating that they can form stable base pairing with the template to produce a recessed 3′ end next to the C nucleotide. Primer oligonucleotides 15-3, 15-2, 15-1, and 15-0 do not label significantly indicating that they cannot form stable base pairing regions with the template. Figure 7C is a graphic representation of the relative labeling intensities of the primer oligonucleotides. The 15-9 primer oligonucleotide labels most intensely indicating that an optimum length or base pairing stability has been reached.

On the right side of Fig. 7B primer oligonucleotides 15-15, 15-12, 15-9, and 15-6 were mixed with template oligonucleotide 30-15-7-1-7-A. Although these oligonucleotides can form the same base pairing as the oligonucleotides on the left side of Fig. 7B, these oligonucleotides do not label because there is not a C nucleotide complementary to GTP next to the 3′ end.

### Interdependence of optimization parameters

**Figure 8:**
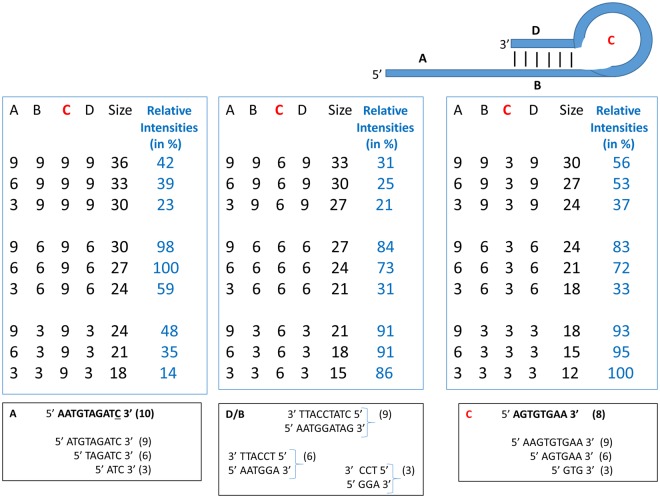
A list of 27 oligonucleotides of the type shown at the top, used to test the interdependence of optimization parameters for 3′ oligonucleotide labeling. Columns denote (**A**) the length of their 5′ extensions, (**C**) the length of their hairpin loops, (**B**,**D**) duplex length, total length (size), and the relative labeling intensities. Labeling intensities are shown as the relative intensities of the set of 27 oligonucleotides where the most intense band (here, in two oligonucleotides) is defined as 100%. Boxes A, B/D, and C are the actual sequences of each length of each part of the oligonucleotides.

While overall oligonucleotide length does not correlate with labeling efficiency, experiments to determine the optimum length of the 5′ extension, the double stranded region (stem), and the loop for the efficiency of labeling the 3′ end with a specific nucleotide give optimum lengths for one of the parameters when the other two parameters are held constant. However, the optimums for a parameter do not appear be independent, since a different optimum for a parameter is observed when the other two parameters are fixed at different lengths. To determine the interdependence of the three parameter lengths, 27 oligonucleotides were created with 3, 6, or 9 nucleotides in the 5′ extension, double stranded region, or loop in all possible combinations [Fig. 8]. The relative labeling intensity of each oligonucleotide is shown.

Analysis of these results allows several generalizations to be made about the interdependence of the parameter lengths. Labeling efficiency generally increases with 5′ extension length for a given stem length/loop length. However, different stem length/loop length combinations give significantly different efficiencies with 3, 6, or 9 nucleotides in the 5′ extension.

The labeling efficiencies generally either decrease with the length of the double stranded stem region (3 base pairs >6 base pairs >9 base pairs) for six 5′ extension/loop combinations or peak with 6 base pairs (6 base pairs >3 or 9 base pairs) for the three 5′ extension/loop combinations with loops of 9 nucleotides.

Labeling efficiencies vary with loop length in several different ways. For the three oligonucleotides with 6 base pair stems, 9 nucleotides loops have the highest labeling efficiency (9 nucleotides >3 or 6 nucleotides). For the three oligonucleotides with 3 base pair stems, 9 nucleotide loops have the lowest efficiency (3 or 6 nucleotides >9 nucleotides). For the remaining three oligonucleotides with 9 base pair stems, 6 nucleotide loops have the lowest labeling efficiency (3 and 9 nucleotides >6 nucleotides or 3 nucleotides >6 or 9 nucleotides).

The highest labeling efficiencies are with oligonucleotides 12-3-3-3-3 and 27-6-6-9-6 followed closely by 30-9-6-9-6, 15-6-3-3-3, and 18-9-3-3-3. Oligonucleotides in the X-X-3-3-3 series have high labeling efficiency, essentially independent of 5′ extension length. Oligonucleotides of the X-X-6-9-6 series have significantly higher labeling efficiencies with 6 or 9 nucleotide (longer) 5′ extensions.

### Labeling efficiency and stem length

Since the formation of a base paired region (stem) is necessary to put a recessed 3′ primer end on the 5′ extension template, it is surprising that longer stem lengths generally give decreased labeling efficiencies. While at least three nucleotides of base pairing complementarity are needed for significant labeling, labeling efficiencies with increasingly longer base pairing potential generally increase up to a peak of about 5 – 7 base pairs and then decline with increasing length, where loop length and 5′ extension length are held constant. This duplex optimum could be caused by at least two possible constraints, [A] a duplex length constraint (static model) in which only duplexes of intermediate length can be bound productively, and [B] a duplex stability constraint (dynamic model) where a duplex is required but where labeling efficiency decreases with duplex stability, indicating that both single stranded and duplex forms are necessary.

Efficiency probably does not depend directly on duplex length since different loop lengths and 5′ extension lengths, when held constant, give a different optimum duplex length (e.g. 9 nucleotides, see Figs 5B and 6B).

**Figure 9:**
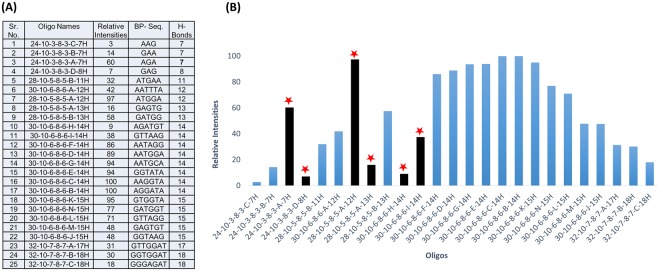
Relative labeling intensities of oligonucleotides with fixed 5′-extension (10 nucleotides), loop length (8 nucleotides) and duplex length (6 nucleotides), but with different duplex sequences. (**A**) A list of the 25 oligonucleotides assayed in Fig. S1 showing [1] the oligonucleotide names, [2] the relative intensities of the 3′ end labeling as determined from the autoradiographs in panels (A) and (B) of Fig. S1, [3] the base pair sequence of the duplex, 5′ to 3′, and [4] the number of hydrogen bonds in the base pairs of the duplex. (B) Graphical representation of the relative labeling intensities of the 25 oligonucleotides from the list in panel (A) plotted against the number of hydrogen bonds in their duplex. The five samples in black with a red star are exceptions to the general trend.

To determine whether duplex stability is a critical factor in labeling efficiency, we assayed 25 different oligonucleotides with constant loop length (8 nucleotides), constant 5′ extension (10 nucleotides), and duplex regions varying from 3 nucleotides to 7 nucleotides and from 7 to 18 hydrogen bonds (Figs 9 and S1). Except for 5 of the 25 nucleotides (with duplex sequences of AGATGT, GAGTG, GTTAAG, AGA, and ATGGA) labeling efficiency increased with hydrogen bond number up to 14 hydrogen bonds and decreased between 14 and 18 hydrogen bonds, consistent with duplex stability being the major factor in labeling efficiency and supporting a dynamic model of labeling (Fig. 9). The five exceptions (shown in black with red stars in Fig. 9B) had both relative labeling efficiencies higher (AGA and ATGGA) and lower (AGATGT, GAGTG, and GTTAAG) than expected for the number of hydrogen bonds in the duplex of the oligonucleotide. In addition, the remaining eight oligonucleotides with 14 hydrogen bonds in its duplex varied in efficiency from 86% to 100%. Likewise, the five oligonucleotides with duplexes with 15 hydrogen bonds varied from 48% to 95% relative labeling efficiency. The five exceptional oligonucleotides and the variation of labeling efficiency in groups of oligonucleotides with the same number of hydrogen bonds indicate that while duplex stability (hydrogen bond number) generally correlates with labeling efficiency, variation within this correlation is observed which may be due to the base composition or to a specific nucleotide sequence motif in the duplex.

In order to determine the cause of the variation in relative labeling efficiency within oligonucleotides with duplexes with the same number of hydrogen bonds, we compared relative labeling efficiencies of oligonucleotides with different base compositions but with the same number of hydrogen bonds in the duplex. There are 15 different base compositions of 6 nucleotides with 14 hydrogen bonds, and there are 16 different base compositions of 6 nucleotides with 15 hydrogen bonds. Three similar six base compositions with 14 hydrogen bonds (A_3_G_2_T, A_3_GCT, A_2_G_2_T_2_) and two similar base composition with 15 hydrogen bonds (AG_3_T and A_2_G_3_T) were compared. The four oligonucleotides with base composition A_3_G_2_T or A_3_GCT varied in relative labeling efficiency from 86% to 100%, and the four oligonucleotides with base composition A_2_G_2_T_2_ varied in relative labeling efficiency from 9% to 100%. Likewise, the four oligonucleotides with base composition AG_3_T varied in relative labeling efficiency from 48–95%. These wide variations in relative labeling efficiency with oligonucleotides with the same base composition in their duplex eliminated base composition as a factor in relative labeling efficiency.

To determine the role of specific sequence motifs as a factor in relative labeling efficiency, we scanned the 13 duplex sequences with 6 base pairs and 14 or 15 hydrogen bonds in the duplex for common motifs which were consistent in relative labeling efficiency. In general, sequences with GG motifs in the duplex labeled more efficiently (48% to 100%) than oligonucleotides in which the G nucleotides in the duplex were separated by other nucleotides (9% to 48%).

## Discussion

This paper describes and characterizes a novel DNA/RNA editing activity of T7 RNAP with nucleotide specificity. This novel activity is detected when an oligonucleotide is provided that has a duplex with a recessed 3′ end (primer) and a 5′ extension (template) where the first unpaired base specifies the labeling nucleotide through Watson-Crick base pairing. This novel editing activity differs from the two classic transcription activities, initiation and elongation. It differs from initiation in that nucleotide addition occurs in the absence of a promoter, and differs from elongation in that nucleotides are not added processively on the template, but only one or a few nucleotides are added since each nucleotide added to the duplex ultimately decreases the labeling efficiency. Probably the most unique feature of this novel activity is the addition of a DNA templated ribonucleotide to a DNA 3′ end to form a DNA primed, RNA-DNA duplex. Although T7 RNAP will only add ribonucleotides to 3′ ends to create RNA strands, it is able to add ribonucleotides to 3′ ends of either RNA or DNA and will use either RNA or DNA templates to specify ribonucleotide addition. Therefore, either RNA or DNA oligonucleotides able to form intramolecular and intermolecular duplexes with recessed 3′ ends can be specifically labeled with radiolabeled ribonucleotides as well as DNA oligonucleotide templates with RNA primers with recessed 3′ ends or RNA oligonucleotide templates with DNA primers with recessed 3′ ends.

There have been a few reports of non-templated nucleotide addition to 3′ ends of nucleic acids with T7 RNAP^57^ and other single subunit RNAPs^20,44,46,47,53–55^. These reports did not mention the potential for nucleotide specificity presumably because they were using complex templates that positioned 3′ recessed ends at many positions, and therefore, next to essentially any nucleotide. Rather than studying this activity, most of the focus was on blocking non-templated nucleotide addition which produced *in vitro* transcripts with unintended sequences. A few studies using less complex templates demonstrated that T7 RNAP could add nucleotides to RNA in the absence of DNA, presumably through inter- and intramolecular formation of RNA-RNA duplexes^48^. Konarska and Sharp^44^ have reported ribonucleotide addition to RNA templated, RNA primed oligonucleotides but did not explore the mechanisms of this activity and did not report activity on DNA templated or DNA primed oligonucleotides. Krupp^45^ observed promoter independent RNA synthesis on DNA templates able to form intermolecular or intramolecular hairpin loops that could be used as DNA primers for RNA synthesis forming RNA-DNA duplexes. However, none of these reports discussed the actual mechanism and criteria for T7 RNAP to add these apparently non DNA-templated nucleotides to the 3′ end. These reactions were all done with four ribonucleotide triphosphates present and often produced complex gel patterns that were complicated to interpret. Here we used DNA oligonucleotides with a single radiolabeled ribonucleotide triphosphate present to simplify interpretation.

To determine their contribution to labeling efficiency, we have systematically varied the three parts of the oligonucleotide (duplex, 5′ extension, loop). In general, labeling efficiency increases with 5′ extension length up to about 10 nucleotides. The loop length can affect labeling efficiency depending on duplex length, but is essentially optional since intermolecular base pairing between two separate oligonucleotides can also produce 3′ end labeling. Small loops when present seem to destabilize a duplex and longer than optimal duplexes can be activated by a small loop while optimal length duplexes may be inactivated by a short loop. On the other hand, longer loops may be detrimental since they increase the distance between the component strands of the duplex decreasing the probability of duplex formation. The most critical portion of these oligonucleotides is the duplex which positions the 3′ end recessed relative to the 5′ end. The duplex length for optimal labeling is about six base pairs for duplexes 33% GC rich (14 hydrogen bonds), about 9 nucleotides at 0% GC (18 hydrogen bonds), or about three base pairs at 66% GC (8 hydrogen bonds).

The observation that duplex stability is a major factor in labeling efficiency implies a dynamic, two-step mechanism for labeling. Although the duplex must transiently form in order to prime for labeling, duplex instability implies that an unfolded single strand intermediate is required for polymerase binding. If the polymerase binds the unfolded single strand oligonucleotide in a productive way, so that the duplex can form with the 3′ end base paired and at the active site of the T7 RNAP, a nucleotide complementary to the template base binds, and a phosphodiester bond is formed incorporating the base into the duplex. In this model, phosphodiester bond formation is coupled to the release of the T7 RNAP. This non-processive mechanism is in contrast to elongation which uses a processive, primer extension on template mechanism. Although duplex stability appears to be a major factor in labeling efficiency, additional sequence motifs may enhance RNA polymerase binding and therefore contribute to labeling efficiency. One motif that seems to enhance 3′ end labeling in oligonucleotides is consecutive G nucleotides (GG motif).

One of the major differences between RNA polymerases and DNA polymerases is the ability of RNAP to initiate synthesis *de novo* by binding to a promoter and then transitioning to elongation through promoter clearance. Early RNAPs probably lacked promoter recognition domains and initiated through primer extension like their closely-related DNA polymerases or through some other non-promoter mechanism. Several contemporary examples of such RNA polymerases exist^20,48,52^. It is likely that the RNA/DNA editing activity of T7 RNAP derives from such a primal activity. Mutations in the gene for T7 RNAP produce altered enzymes which support this idea. For example, some mutated enzymes have been found to be catalytically active although they bind promoter weakly, and in some instances mutated enzymes which are transcriptionally inactive have strong binding to the promoter^58^. Also, mutated T7 RNA polymerase^59^ with two amino acid substitutions (Y639F and S641A) has altered specificity towards promoter, but gains the ability to utilize dNTPs and catalyze RNA and DNA synthesis from circular supercoiled plasmid DNA^60^. This Y639F mutant retains the ability to use RNA or DNA templates and can display de novo initiated or primed DNA-directed DNA polymerase, reverse transcriptase, RNA-directed RNA polymerase or DNA-directed RNA polymerase activities depending simply on the templates and substrates presented to it in the synthesis reaction^55^.

This novel DNA/RNA editing activity in T7 RNAP is probably present in all single subunit polymerases and may be the basis of nucleotide specificity during RNA editing by the mtRNAP in mitochondria of myxomycetes^61^. One of the major questions about Myxomycete RNA editing is how the identity of the inserted nucleotide is determined. Based on the observed *in vitro* mechanism of editing in T7 RNAP, we propose a model of nucleotide specificity in RNA editing for the related *Physarum* mtRNAP. At an RNA editing site, the *Physarum* mtRNAP pauses and then may either stay in place, release, or back up on the template to allow the 3′ end of the RNA to loop back on a complementary sequence either in the RNA or in the non-template strand of DNA, and add a nucleotide at the 3′ end. This positioning of the 3′ end allows specific addition to the 3′ end using the vacant position next to the 3′ end to specify the added nucleotide through Watson-Crick base pairing. The transient base pairing of the RNA is then released resulting in the insertion of a nucleotide at the 3′ end not templated by the template strand of DNA. Elongation is then resumed as a phosphodiester bond is formed between the unpaired 3′ nucleotide and the next templated nucleotide. *In vitro* presentation of the *Physarum* mitochondrial RNAP (mtRNAP) with oligonucleotides, similar to those described above, would be a test of this hypothesis. Confirmation of this novel activity in *Physarum* mtRNAP would be a major step toward understanding the co-transcriptional mechanism of RNA editing by the mtRNAP in mitochondria of myxomycetes.

## Electronic supplementary material

Supplementary Information
