# Supplementary material for: A specific, promoter-independent activity of T7 RNA polymerase suggests a general model for DNA/RNA editing in single subunit RNA Polymerases
Source: Sci Rep. 2018 Sep 17;8:13885. doi: 10.1038/s41598-018-32231-6 (PMC6141538; doi:10.1038/s41598-018-32231-6)
Supplement: Supplementary file 1 — Supplementary Information [file 41598_2018_32231_MOESM1_ESM.pdf]

A specific, promoter-independent activity of T7 RNA polymerase suggests a general model for DNA/RNA editing in single subunit RNA Polymerases

Short title: DNA and RNA editing by T7 RNA polymerase

Subha Narayan Sarcar[1] and Dennis L Miller[1,*]

[1] Department of Biological Sciences, The University of Texas at Dallas, Richardson, Texas, 75083-0688, USA

* To whom correspondence should be addressed. Tel: +1 (972) 883-2539; Fax: (972) 883-2409; Email: dmiller@utdallas.edu

Table S1

Sequences of single stranded oligonucleotides used in the experiments in this paper, organized by Fig and showing the oligonucleotide lengths and sequences of the duplex region.

Serial No.	Names on Paper	Oligos for Figure-2	bp region
1	36-6-6-18-6-A	5’GGATTTAATCCAGATATGATAGAGTGTGAATGGATT 3’	AATCCA
2	36-6-6-18-6-G	5’GGATTCAATCCAGATATGATAGAGTGTGAATGGATT 3’	AATCCA
		**Oligos for Figure-3**	
4	36-16-6-8-6	5’GGATTGAATGTAGATCAATGGAAGTGTGAATCCATT 3’	AATGGA
5	30-10-6-8-6	5’ AATGTAGATCAATGGAAGTGTGAATCCATT 3’	AATGGA
6	25-5-6-8-6	5’ AGATCAATGGAAGTGTGAATCCATT 3’	AATGGA
7	23-3-6-8-6	5’ CCCAATGGAAGTGTGAATCCATT 3’	AATGGA
8	22-2-6-8-6	5’ CCAATGGAAGTGTGAATCCATT 3’	AATGGA
9	21-1-6-8-6	5’ CAATGGAAGTGTGAATCCATT 3’	AATGGA
10	20-0-6-8-6	5’ AATGGAAGTGTGAATCCATT 3’	AATGGA
		**Oligos for Figure-4**	
11	20-2-6-6-6	5’TCTATAATATAAGAATTATA 3’	TATAAT
12	23-2-6-9-6	5’TCTATAATATAAGATTGATTATA 3’	TATAAT
13	26-2-6-12-6	5’TCTATAATATTATAAGATTGATTATA 3’	TATAAT
14	29-2-6-15-6	5’TCTATAATTATTATAAGATTGTTATTATA 3’	TATAAT
15	32-2-6-18-6	5’TCTATAATATTATTATAAGATTGTTAATTATA 3’	TATAAT
16	35-2-6-21-6	5’TCTATAATTATTATTATAAGATTGTTATAATTATA 3’	TATAAT
17	38-2-6-24-6	5’TCTATAATAATATTATTATAAGATTGTTATAGATTATA 3’	TATAAT
18	26-2-9-6-9	5’TCTATAATATTAGATTGAATATTATA 3’	TATAATATT
19	29-2-9-9-9	5’TCTATAATATTATAAGATTGAATATTATA 3’	TATAATATT
20	32-2-9-12-9	5’TCTATAATATTATTATAAGATTGAATATTATA 3’	TATAATATT
21	35-2-9-15-9	5’TCTATAATATTTATTATAAGATTGTTAATATTATA 3’	TATAATATT
22	38-2-9-18-9	5’TCTATAATATTATTATTATAAGATTGTTAAATATTATA 3’	TATAATATT
23	41-2-9-21-9	5’TCTATAATATTTATTATTATAAGATTGTTATAAATATTATA 3’	TATAATATT
24	44-2-9-24-9	5’TCTATAATATTAATATTATTATAAGATTGTTATAGAATATTATA 3’	TATAATATT
		**Oligos for Figure-5**	
25	32-2-15-0-15	5’TCTATAATATTATTATA TATAATAATATTATA 3’	TATAATATTATTATA
26	32-2-12-6-12	5’TCTATAATATTATTATAAGAAATAATATTATA 3’	TATAATATTATT
27	32-2-9-12-9	5’TCTATAATATTATTATAAGATTGAATATTATA 3’	TATAATATT
28	32-2-6-18-6	5’TCTATAATATTATTATAAGATTGTTAATTATA 3’	TATAAT
29	32-2-3-24-3	5’TCTATAATATTATTATAAGATTGTTATAGATA 3’	TAT
30	32-2-2-26-2	5’TCTATAATATTATTATAAGATTGTTATAGGTA 3’	TA
31	32-2-1-28-1	5’TCTATAATATTATTATAAGATTGTTATAGGAA 3’	T
32	32-2-0-30-0	5’TCTATAATATTATTATAAGATTGTTATAGGAT 3’	0

Serial No.	Names on Paper	Oligos for Figure-6	bp region
33	30-10-6-8-6	5’ AATGTAGATCAATGGAAGTGTGAATCCATT 3’	AATGGA
34	28-10-5-8-5	5’ AATGTAGATCATGGAAGTGTGAATCCAT 3’	ATGGA
35	26-10-4-8-4	5’ AATGTAGATCTGGAAGTGTGAATCCA 3’	TGGA
36	24-10-3-8-3	5’ AATGTAGATCGGAAGTGTGAATCC 3’	GGA
37	22-10-2-8-2	5’ AATGTAGATCGAAGTGTGAATC 3’	GA
38	20-10-1-8-1	5’ AATGTAGATCAAGTGTGAAT 3’	A
39	18-10-0-8-0	5’ AATGTAGATCAGTGTGAA 3’	0
40	20-2-3-12-3	5’TCTATATTATA·AGATTGATA 3’	TAT
41	26-2-6-12-6	5’TCTATAATATTATA·AGATTGATTATA 3’	TATAAT
42	28-2-7-12-7	5’TCTATAATAATTATAAGATTGTATTATA 3’	TATAATA
43	30-2-8-12-8	5’TCTATAATATATTATAAGATTGATATTATA 3’	TATAATAT
44	32-2-9-12-9	5’TCTATAATATTATTATAAGATTGAATATTATA 3’	TATAATATT
45	34-2-10-12-10	5’TCTATAATATTAATTATAAGATTGTAATATTATA 3’	TATAATATTA
46	36-2-11-12-11	5’TCTATAATATTATATTATAAGATTGATAATATTATA 3’	TATAATATTAT
47	38-2-12-12-12	5’TCTATAATATTATTATTATAAGATTGAATAATATTATA 3’	TATAATATTATT
48	44-2-15-12-15	5’TCTATAATATTATTATAATTATAAGATTGTATAATAATATTATA 3’	TATAATATTATTATA
49	50-2-18-12-18	5’TCTATAATAATATTATTATAATTATAAGATTGTATAATAATATTATTATA 3’	TATAATAATATTATTATA
		**Oligos for Figure-7**	
50	45-15-6-18-6	5’GATTGAATGTAGATCTATAATATTATTATAAGATTGTTAATTATA 3’	TATAAT
51	30-15-7-1-7-A	5’GATTGAATGTAGATTTATAATATTATTATA 3’	TATAATA
52	30-15-7-1-7-G	5’GATTGAATGTAGATCTATAATATTATTATA 3’	TATAATA
53	15-15	3’ATATTATAATAATAT 5’	ATATTATAATAATAT
54	15-12	3’ATATTATAATAAAGA 5’	ATATTATAATAA
55	15-9	3’ATATTATAAGTTAGA 5’	ATATTATAA
56	15-6	3’ATATTAATTGTTAGA 5’	ATATTA
57	15-3	3’ATAGATATTGTTAGA 5’	ATA
58	15-2	3’ATGGATATTGTTAGA 5’	AT
59	15-1	3’AAGGATATTGTTAGA 5’	A
60	15-0	3’TAGGATATTGTTAGA 5’	0

Serial No.	Names on Paper	Oligos for Figure-8	bp region
61	36-9-9-9-9	5' ATGTAGATCAATGGATAGAAGTGTGAACTATCCATT 3'	AATGGATAG
62	33-6-9-9-9	5' TAGATCAATGGATAGAAGTGTGAACTATCCATT 3'	AATGGATAG
63	30-3-9-9-9	5' ATCAATGGATAGAAGTGTGAACTATCCATT 3'	AATGGATAG
64	30-9-6-9-6	5' ATGTAGATCAATGGAAAGTGTGAATCCATT 3'	AATGGA
65	27-6-6-9-6	5' TAGATCAATGGAAAGTGTGAATCCATT 3'	AATGGA
66	24-3-6-9-6	5' ATCAATGGAAAGTGTGAATCCATT 3'	AATGGA
67	24-9-3-9-3	5' ATGTAGATCGGAAAGTGTGAATCC 3'	GGA
68	21-6-3-9-3	5' TAGATCGGAAAGTGTGAATCC 3'	GGA
69	18-3-3-9-3	5' ATCGGAAAGTGTGAATCC 3'	GGA
70	33-9-9-6-9	5' ATGTAGATCAATGGATAGAGTGAACTATCCATT 3'	AATGGATAG
71	30-6-9-6-9	5' TAGATCAATGGATAGAGTGAACTATCCATT 3'	AATGGATAG
72	27-3-9-6-9	5' ATCAATGGATAGAGTGAACTATCCATT 3'	AATGGATAG
73	27-9-6-6-6	5' ATGTAGATCAATGGAAGTGAATCCATT 3'	AATGGA
74	24-6-6-6-6	5' TAGATCAATGGAAGTGAATCCATT 3'	AATGGA
75	21-3-6-6-6	5' ATCAATGGAAGTGAATCCATT 3'	AATGGA
76	21-9-3-6-3	5' ATGTAGATCGGAAGTGAATCC 3'	GGA
77	18-6-3-6-3	5' TAGATCGGAAGTGAATCC 3'	GGA
78	15-3-3-6-3	5' ATCGGAAGTGAATCC 3'	GGA
79	30-9-9-3-9	5' ATGTAGATCAATGGATAGGTGCTATCCATT 3'	AATGGATAG
80	27-6-9-3-9	5' TAGATCAATGGATAGGTGCTATCCATT 3'	AATGGATAG
81	24-3-9-3-9	5' ATCAATGGATAGGTGCTATCCATT 3'	AATGGATAG
82	24-9-6-3-6	5' ATCAATGGATAGGTGCTATCCATT 3'	AATGGA
83	21-6-6-3-6	5' TAGATCAATGGAGTGTCCATT 3'	AATGGA
84	18-3-6-3-6	5' ATCAATGGAGTGTCCATT 3'	AATGGA
85	18-9-3-3-3	5' ATGTAGATCGGAGTGTCC 3'	GGA
86	15-6-3-3-3	5' TAGATCGGAGTGTCC 3'	GGA
87	12-3-3-3-3	5' ATCGGAGTGTCC 3'	GGA

Oligos used in Fig 9 are same, as used in Fig S1-A and Fig S1-B

Serial No.	Names on Paper	Oligos for Figure-S1-A	bp region
88	24-10-3-8-3-A-7H	5' AATGTAGATCAGAAGTGTGAATCT 3'	AGA
89	24-10-3-8-3-B-7H	5' AATGTAGATCGAAAGTGTGAATTC 3'	GAA
90	24-10-3-8-3-C-7H	5' AATGTAGATCAAGAGTGTGAACTT 3'	AAG
91	24-10-3-8-3-D-8H	5' AATGTAGATCGAGAGTGTGAACTC 3'	GAG
92	28-10-5-8-5-A-12H	5' AATGTAGATCATGGAAGTGTGAATCCAT 3'	ATGGA
93	28-10-5-8-5-B-11H	5' AATGTAGATCATGAAAGTGTGAATTCAT 3'	ATGAA
94	30-10-6-8-6-A-12H	5' AATGTAGATCAATTTAAGTGTGAATAAATT 3'	AATTTA
95	30-10-6-8-6-B-14H	5' AATGTAGATCAGGATAAGTGTGAATATCCT 3'	AGGATA
96	30-10-6-8-6-C-14H	5' AATGTAGATCAAGGTAAGTGTGAATACCTT 3'	AAGGTA
97	30-10-6-8-6-D-14H	5' AATGTAGATCAATGGAAGTGTGAATCCATT 3'	AATGGA
98	28-10-5-8-5-A-13H	5' AATGTAGATCGAGTGAGTGTGAACACTC 3'	GAGTG
99	28-10-5-8-5-B-13H	5' AATGTAGATCGATGGAGTGTGAACCATC 3'	GATGG

Serial No.	Names on Paper	Oligos for Figure-S1-B	bp region
100	30-10-6-8-6-D-14H	5' AATGTAGATCAATGGAAGTGTGAATCCATT 3'	AATGGA
101	30-10-6-8-6-E-14H	5' AATGTAGATCGGTATAAGTGTGAATATACC 3'	GGTATA
102	30-10-6-8-6-C-14H	5' AATGTAGATCAAGGTAAGTGTGAATACCTT 3'	AAGGTA
103	30-10-6-8-6-F-14H	5' AATGTAGATCAATAGGAGTGTGAACCTATT 3'	AATAGG
104	30-10-6-8-6-G-14H	5' AATGTAGATCAATGCAAGTGTGAATGCATT 3'	AATGCA
105	30-10-6-8-6-H-14H	5' AATGTAGATCAGATGTAGTGTGAATACCTT 3'	AGATGT
106	30-10-6-8-6-I-14H	5' AATGTAGATCGTTAAGAGTGTGAACTTAAC 3'	GTTAAG
107	30-10-6-8-6-J-15H	5' AATGTAGATCGGTAAGAGTGTGAACTTACC 3'	GGTAAG
108	30-10-6-8-6-K-15H	5' AATGTAGATCGTGGTAAGTGTGAATACCAC 3'	GTGGTA
109	30-10-6-8-6-L-15H	5' AATGTAGATCGTTAGGAGTGTGAACCTAAC 3'	GTTAGG
110	30-10-6-8-6-M-15H	5' AATGTAGATCGAGTGTAGTGTGAAACACTC 3'	GAGTGT
111	30-10-6-8-6-N-15H	5' AATGTAGATCGATGGTAGTGTGAAACCATC 3'	GATGGT
112	32-10-7-8-7-A-17H	5' AATGTAGATCGTTGGATAGTGTGAAATCCAAC 3'	GTTGGAT
113	32-10-7-8-7-B-18H	5' AATGTAGATCGGTGGATAGTGTGAAATCCACC 3'	GGTGGAT
114	32-10-7-8-7-C-18H	5' AATGTAGATCGGGAGATAGTGTGAAATCTCCC 3'	GGGAGAT

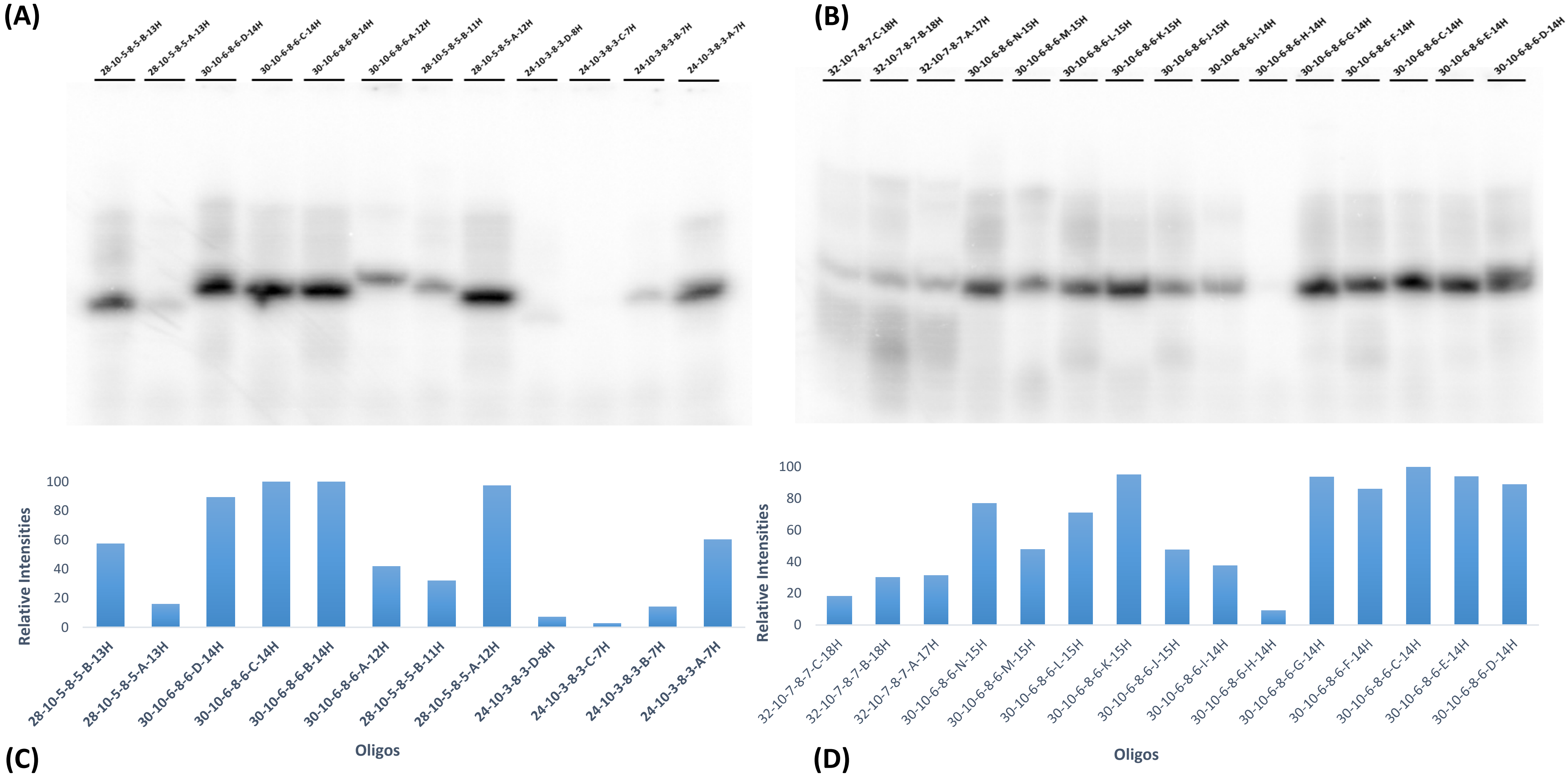

Fig S1. **Effect of duplex stability (number of duplex hydrogen bonds) and duplex sequence variation on relative 3’ end labeling efficiency.**
Panels (A) and (B) are autoradiographs of 15% polyacrylamide gels which show the 3’ end labeling intensities for oligonucleotides with constant 5’ extension (10 nucleotides) and constant loop length (8 nucleotides) but with varying duplex length, hydrogen bond number and sequence. Panel (C) and (D) are graphical representations showing the relative intensities of oligonucleotide labeling measured in percentage intensity of the most intensely labeled gel band.
